# A large-scale observational study linking various kinds of physical exercise to lipoprotein-lipid profile

**DOI:** 10.1186/s12970-021-00436-2

**Published:** 2021-05-10

**Authors:** Wan-Yu Lin

**Affiliations:** 1 Institute of Epidemiology and Preventive Medicine, College of Public Health, National Taiwan University, Room 501, No. 17, Xu-Zhou Road, Taipei, 100 Taiwan; 2 Department of Public Health, College of Public Health, National Taiwan University, Room 501, No. 17, Xu-Zhou Road, Taipei, 100 Taiwan

**Keywords:** High-density lipoprotein cholesterol, Jogging, Low-density lipoprotein cholesterol, Sport, Triglyceride

## Abstract

**Background:**

Being a major cardiovascular risk factor, dyslipidemia is a critical problem in public health. Recommendations in performing regular physical exercise are important to prevent dyslipidemia.

**Methods:**

Based on a discovery cohort with 27,735 subjects and a replication cohort with 67,512 subjects, we evaluated the associations of regularly performing 23 exercises with 4 dyslipidemia indices measured from serum, including triglyceride (TG), low-density lipoprotein cholesterol (LDL-C), high-density lipoprotein cholesterol (HDL-C), and TG/HDL-C ratio. Regular exercise was defined as performing 30 min of “exercise” three times a week. “Exercise” includes leisure-time activities such as jogging, swimming, etc. Sex, age, body mass index, alcohol drinking, cigarette smoking, and education level were adjusted in all statistical analyses.

**Results:**

Among the 23 exercises, only jogging was associated with a decreased level of TG (95% confidence interval [C.I.] = 5.9–14.5 mg/dL) and TG/HDL-C ratio (95% C.I. = 0.22–0.49). A total of 5 exercises were associated with an increased level of HDL-C, including jogging (95% C.I. = 2.1–3.3 mg/dL), swimming (95% C.I. = 1.6–3.3 mg/dL), dance dance revolution (95% C.I. = 1.5–3.4 mg/dL), international standard dancing (95% C.I. = 1.0–2.7 mg/dL), and cycling (95% C.I. = 0.6–1.8 mg/dL). These significant findings were further well replicated in the cohort of 67,512 subjects.

**Conclusion:**

Regular jogging was not only associated with an increased level of HDL-C, but also the only one exercise associated with a decreased level of TG and TG/HDL-C ratio. Nonetheless, jogging may be difficult to engage in for subjects with limited exercise capacity. We here found that swimming, dancing, and cycling are also significantly associated with an increased level of HDL-C. People who are seeking exercise to improve their lipoprotein-lipid profiles can have other choices now.

## Background

Dyslipidemia is an unhealthy level of lipids in blood, including elevated triglyceride (TG) or low-density lipoprotein cholesterol (LDL-C), and decreased high-density lipoprotein cholesterol (HDL-C). Moreover, the ratio of TG to HDL-C (TG/HDL-C ratio) is a useful indicator to detect metabolic syndrome [1].

Around ~ 25% of people in the United States are living with hypertriglyceridemia (TG > 150 mg/dL), which is a type of dyslipidemia and is associated with an increased risk of cardiovascular diseases [2]. According to the Ministry of Health and Welfare in Taiwan, a TG level > 150 mg/dL; an LDL-C level > 130 mg/dL; a HDL-C level < 40 mg/dL for males or a HDL-C level < 50 mg/dL for females, are considered dyslipidemia. As a major cardiovascular risk factor, dyslipidemia is a critical problem in public health. Lifestyle modification such as performing physical exercise is a low-cost and non-pharmacological strategy to improve one’s lipoprotein-lipid profile.

Studies have shown that TG decreased and HDL-C increased after 24 h of aerobic exercise training, and lasted through 48 h [3]. Some studies found that HDL-C is more sensitive to aerobic exercise than TG and LDL-C [4]. Regular exercise increases the level of HDL-C in a dose-response manner [5–8]. Almost all studies indicated that exercise increases HDL-C to some degree, which is a consistent result from both human [4, 9–13] and rats [14–16]. Moreover, regular exercise was found to improve “cholesterol efflux capacity”, the ability of HDL-C to carry cholesterol from one’s bloodstream to the liver for clearance [17].

Different from HDL-C, exercise effects on LDL-C have been inconsistent across studies. Some studies even provided completely contradictory results [4, 9–12]. It has been suggested that exercise alone may not lower LDL-C, unless a loss of body weight is also achieved in this period of time [18]. Other studies concluded that, compared with HDL-C, exercise with a higher intensity is required to reduce the level of LDL-C and TG [19].

Despite many randomized controlled trials (RCTs) related to this topic, the investigated aerobic exercises were limited to walking, cycling, or jogging [7, 9–12, 20, 21]. Very few studies have evaluated the effects of other aerobic exercises such as dancing, mountain climbing, swimming, playing tennis, etc. Moreover, it remains unknown which exercises are more related to a lower risk of dyslipidemia.

To address this issue, we applied data of 95,247 Taiwan Biobank (TWB) participants to evaluate the associations of regular exercise with their TG, LDL-C, HDL-C, and TG/HDL-C levels. The 95,247 TWB subjects were genotyped by one of two single-nucleotide polymorphism arrays. Therefore, by nature, we separated them into a discovery cohort and a replication cohort according to their genotyping arrays.

In this study, we aim to identify exercises that are associated with a lower risk of dyslipidemia according to the discovery cohort, and then we seek replication from the replication cohort. While most previous studies were RCTs with limited sample sizes and exercise kinds [7, 9–12, 21], this work is an observational study with a much larger sample size and more than 20 kinds of exercise.

## Methods

### Taiwan biobank

From October 2012 to February 2020, TWB recruited 95,247 Taiwan residents aged 30 to 70 years. Among the 95,247 subjects, 27,735 and 67,512 subjects were whole-genome genotyped by the TWB1 and TWB2 genotyping arrays, separately. They were so-called the TWB1 and TWB2 cohorts, respectively [22]. Although the current study includes no genetic analysis, the two cohorts formed a natural separation of a discovery set and a replication set. We here used the 27,735 subjects genotyped by the TWB1 array as the discovery cohort and the 67,512 subjects genotyped by the TWB2 array as the replication cohort. After signing informed consent, these 95,247 community-based volunteers provided blood and urine samples, took physical examinations, and reported lifestyle factors (regular exercise, alcohol drinking, and cigarette smoking) through a face-to-face interview with TWB researchers [23].

TWB was approved by the Institutional Review Board on Biomedical Science Research/IRB-BM, Academia Sinica, and also by the Ethics and Governance Council of Taiwan Biobank, Taiwan. Written informed consent was obtained before data collection, from each participant in accordance with institutional requirements and the principles of the Declaration of Helsinki. Our use of the TWB research data was approved by TWB on February 18, 2020 (application number: TWBR10810–07). This study further received approval from the Research Ethics Committee of National Taiwan University Hospital (NTUH-REC no. 201805050RINB).

### Dyslipidemia indices

We analyzed 3 indices that are related to dyslipidemia: TG, LDL-C, and HDL-C, all measured from serum. HDL-C is considered as “good cholesterol” in the sense that a higher level of HDL-C is linked to a lower risk of coronary heart disease [24]. On the contrary, LDL-C is usually regarded as the “bad” type of cholesterol [25]. A TG level < 150 mg/dL, an LDL-C level < 130 mg/dL, and a HDL-C level > 40 mg/dL for males or a HDL-C level > 50 mg/dL for females, are considered desirable [26]. In addition to these 3 dyslipidemia indices, we also investigated TG/HDL-C ratio, which is a useful indicator of metabolic syndrome [1]. An elevated TG/HDL-C ratio is strongly associated with an increased risk of adverse cardiac events [27].

### Covariates under adjustment

Sex, age, obesity, alcohol drinking, cigarette smoking, and education level are closely related to dyslipidemia [28]. Therefore, our regression model adjusted sex, age (in years), body mass index (BMI), drinking status (yes vs. no), smoking status (yes vs. no), and educational attainment (a value ranging from 1 to 7). In TWB, drinking was defined as a subject having a weekly intake of more than 150 mL of alcohol for at least 6 months and having not stopped drinking at the time his/her phenotypes were measured. Smoking was defined as a subject who had smoked for at least 6 months and had not quit smoking at the time his/her phenotypes were measured.

Educational attainment was recorded according to a face-to-face interview with TWB researchers, ranging from 1 to 7: 1 “illiterate”, 2 “no formal education but literate”, 3 “primary school graduate”, 4 “junior high school graduate”, 5 “senior high school graduate”, 6 “college graduate”, and 7 “Master’s or higher degree”.

Regular exercise was defined as performing 30 min of “exercise” three times a week. “Exercise” includes leisure-time activities such as jogging, swimming, dancing, cycling, mountain climbing, weight training, etc. Individuals with regular exercise would then be asked what kinds of exercise they usually engaged in.

### Statistical analysis

To investigate exercise effects on the 4 dyslipidemia indices, we considered the following regression model:

1 $$ {Y}_k={\beta}_0+{\sum}_{j=1}^{23}{\beta}_{E_{j,k}}{E}_j+{\sum}_{v=1}^6{\beta}_{C_v}{Covariate}_v+\varepsilon, $$

where *Y*_*k*_ is dyslipidemia index *k* (*k* = 1, 2, 3, or 4), *E*_*j*_ is an indicator variable taking a value of 1 or 0 (*E*_*j*_ = 1 if the subject chose exercise *j* as the regular exercise, and *j* = 1, ⋯, 23), *Covariate*_*v*_ is the *v*^th^ covariate (*v* = 1, ⋯, 6), and *ε* is the random error term. Exercise *j* will be claimed to exhibit effects on index *k* if the *p*-value of ${H}_0:{\beta}_{E_{j,k}}=0$ vs. ${H}_1:{\beta}_{E_{j,k}}\ne 0$ is less than $\frac{0.05}{\left(30\times 4\right)}=0.0004$, which is the Bonferroni-corrected significance level considering 30 regression coefficients (${\beta}_0,{\beta}_{E_{j,k}}, and\ {\beta}_{C_v}$) in model (1) and 4 dyslipidemia indices. All analyses were performed using R version 4.0.3.

## Results

### Basic characteristics of the TWB subjects

Among the 95,247 TWB subjects, 27,735 were classified into the discovery cohort whereas 67,512 the replication cohort. Table 1 presents the basic characteristics of these two cohorts, stratified by sexes. The discovery (replication) cohort shows that 28.6% (29.6%) males and 13.8% (15.9%) females had TG > 150 mg/dL; 38.3% (36.4%) males and 33.6% (35.3%) females had LDL-C > 130 mg/dL; 22.7% (23.2%) males had HDL-C < 40 mg/dL and 27.6% (27.8%) females had HDL-C < 50 mg/dL. The replication cohort presents similar results (Table 1).

**Table 1:** Basic characteristics of the 95,247 Taiwan Biobank subjects

	Males		Females	
	Discovery cohort	Replication cohort	Discovery cohort	Replication cohort
Total	13,834 (49.9)	20,763 (30.8)	13,901 (50.1)	46,749 (69.2)
Age (years)	48.8 ± 11.2	50.8 ± 11.3	48.8 ± 11.1	50.3 ± 10.5
BMI (kg/m^2^)	25.2 ± 3.5	25.4 ± 3.5	23.5 ± 3.7	23.6 ± 3.7
Drinking	1714 (12.4)	2740 (13.2)	246 (1.8)	841 (1.8)
Smoking	2886 (20.9)	4351 (21.0)	397 (2.9)	1334 (2.9)
Educational attainment	5.7 ± 0.9	5.7 ± 0.9	5.3 ± 1.0	5.3 ± 1.0
TG (mg/dL)	133.8 ± 104.5	140.3 ± 127.8	99.4 ± 68.6	103.4 ± 73.8
TG > 150 mg/dL	3951 (28.6)	6151 (29.6)	1916 (13.8)	7434 (15.9)
LDL-C (mg/dL)	122.3 ± 31.4	121.0 ± 31.3	119.4 ± 31.9	120.6 ± 31.8
LDL-C > 130 mg/dL	5295 (38.3)	7555 (36.4)	4676 (33.6)	16,488 (35.3)
HDL-C (mg/dL)	47.9 ± 11.0	47.8 ± 11.1	58.0 ± 13.1	58.1 ± 13.2
HDL-C < 40 mg/dL (males)	3137 (22.7)	4815 (23.2)	–	–
HDL-C < 50 mg/dL (females)	–	–	3837 (27.6)	12,980 (27.8)
TG/HDL-C	3.2 ± 3.4	3.3 ± 4.4	1.9 ± 1.9	2.0 ± 2.1
TG/HDL-C > 3.75 (males) ^a^	3572 (25.8)	5622 (27.1)	–	–
TG/HDL-C > 3 (females) ^a^	–	–	2093 (15.1)	7856 (16.8)

Data are shown in *n* (%) or mean ± SD

^a^Combining TG and HDL-C, the undesirable range for TG/HDL-C is > 3.75 (= 150/40, for male) or > 3 (= 150/50, for female)

To assess exercise effects while adjusting for covariates, we fitted model (1) for the 4 dyslipidemia indices, respectively. The results were presented in Tables 2 and 3. We used the “car” package (https://cran.r-project.org/web/packages/car/) to compute variance inflation factor (VIF). The VIFs under 8 models (4 dyslipidemia indices in 2 cohorts) were all smaller than 2. This suggests that multicollinearity between variables in model (1) is acceptable.

**Table 2:** Associations of 6 covariates with each dyslipidemia index

	TG (mg/dL)		LDL-C (mg/dL)		HDL-C (mg/dL)		TG/HDL-C	
Explanatory variables in model (1)	$\hat{\boldsymbol{\beta}}$	***P***-value	$\hat{\boldsymbol{\beta}}$	***P***-value	$\hat{\boldsymbol{\beta}}$	***P***-value	$\hat{\boldsymbol{\beta}}$	***P***-value
Sex (1: female vs. 0: male)	−18.5 (− 19.1)	2.8E-56 (1.6E-107)	− 1.1 (1.7)	0.008 (3.0E-8)	8.2 (8.3)	0 (0)	−0.77 (− 0.82)	2.6E-99 (1.6E-190)
Age (in years, continuous variable)	0.5 (0.7)	1.6E-23 (1.2E-69)	0.3 (0.4)	7.7E-66 (2.4E-212)	0.01 (0.01)	0.13 (0.04)	0.01 (0.01)	3.5E-9 (4.1E-20)
Body mass index (in kg/m^2^, continuous variable)	6.3 (5.9)	0 (0)	1.3 (1.2)	6.2E-140 (5.5E-276)	−1.1 (−1.2)	0 (0)	0.19 (0.17)	0 (0)
Drinking status (1: yes vs. 0: no)	19.3 (23.6)	9.7E-21 (8.2E-48)	−7.0 (−5.8)	2.6E-20 (4.3E-25)	4.0 (4.4)	6.2E-48 (2.6E-95)	0.26 (0.36)	4.9E-5 (1.2E-11)
Smoking status (1: yes vs. 0: no)	25.7 (31.4)	5.5E-52 (2.0E-119)	2.2 (2.0)	0.0004 (2.2E-5)	−2.9 (−3.1)	4.7E-38 (7.6E-68)	0.83 (1.05)	8.5E-57 (4.1E-127)
Educational attainment (a value ranging from 1 to 7)	−2.7 (−1.6)	3.5E-6 (2.7E-5)	0.4 (0.5)	0.05 (5.0E-5)	0.2 (0.1)	0.02 (0.01)	−0.07 (− 0.05)	9.0E-5 (1.7E-4)
R-square of model (1)	**12.8% (10.9%)**		**4.2% (3.8%)**		**26.5% (24.2%)**		**12.9% (10.0%)**	

Data are given for the discovery cohort (replication cohort)

**Table 3:** Association of performing an exercise with each dyslipidemia index

					TG (mg/dL)		LDL-C (mg/dL)		HDL-C (mg/dL)		TG/HDL-C	
	No. of subjects	% of males	Age (years), mean	Age (years), s.d.	${\hat{\beta}}_{E_{j,1}}$	***P***-value	${\hat{\beta}}_{E_{j,2}}$	***P***-value	${\hat{\beta}}_{E_{j,3}}$	***P***-value	${\hat{\beta}}_{E_{j,4}}$	***P***-value
Walking	3852 (10,185)	46.8 (29.6)	55.8 (56.7)	9.4 (8.4)	−2.05 (− 0.78)	1.8E-01 (4.4E-01)	− 0.67 (− 0.84)	2.4E-01 (1.6E-02)	−0.07 (− 0.26)	7.2E-01 (4.5E-02)	− 0.07 (0.00)	1.2E-01 (1.0E+ 00)
Exercise walking	2152 (6251)	51.8 (31.9)	54.7 (55.1)	9.5 (9.0)	−3.28 (− 2.25)	9.1E-02 (6.7E-02)	− 2.26 (− 0.95)	1.6E-03 (2.6E-02)	− 0.36 (0.60)	1.7E-01 (1.9E-04)	− 0.08 (− 0.05)	2.0E-01 (1.7E-01)
Jogging	1700 (3223)	80.1 (61.5)	45.0 (46.1)	10.2 (10.5)	**−10.20 (− 10.58)**	**3.6E-06 (4.4E-10)**	− 0.30 (− 1.49)	7.1E-01 (1.1E-02)	**2.72 (2.51)**	**2.5E-20 (6.4E-30)**	**−0.36 (− 0.35)**	**1.6E-07 (3.1E-10)**
Cycling	1445 (2620)	68.7 (49.4)	51.2 (52.3)	10.6 (10.5)	−3.29 (−4.69)	1.5E-01 (1.0E-02)	−2.88 (− 2.11)	7.4E-04 (7.8E-04)	**1.17 (1.14)**	**1.7E-04 (1.6E-06)**	−0.12 (− 0.15)	8.5E-02 (1.1E-02)
Mountain climbing	944 (2426)	58.6 (40.0)	55.6 (56.3)	8.4 (7.8)	−6.70 (−4.94)	1.8E-02 (8.9E-03)	−2.16 (0.29)	3.9E-02 (6.5E-01)	0.59 (0.46)	1.2E-01 (6.2E-02)	−0.19 (− 0.14)	3.2E-02 (1.8E-02)
Stretching exercise	852 (2277)	33.9 (17.6)	58.3 (58.6)	8.6 (7.6)	−5.84 (1.21)	5.0E-02 (5.3E-01)	−0.65 (− 0.73)	5.6E-01 (2.8E-01)	0.75 (− 0.06)	5.8E-02 (8.1E-01)	− 0.17 (0.06)	7.1E-02 (3.3E-01)
Yoga	607 (2026)	10.5 (5.6)	51.2 (52.4)	10.4 (9.1)	−3.00 (−3.75)	4.0E-01 (7.2E-02)	0.90 (0.04)	4.9E-01 (9.5E-01)	1.46 (1.03)	2.1E-03 (1.5E-04)	−0.10 (− 0.09)	3.5E-01 (2.0E-01)
International standard dancing	672 (1888)	14.0 (6.4)	57.2 (57.8)	7.9 (6.3)	0.48 (−2.39)	8.9E-01 (2.6E-01)	3.24 (1.54)	8.5E-03 (3.6E-02)	**1.87 (1.58)**	**2.8E-05 (1.1E-08)**	−0.04 (− 0.08)	7.1E-01 (2.2E-01)
Dance dance revolution	598 (1816)	9.7 (5.7)	50.0 (50.9)	11.0 (9.8)	−2.22 (−4.95)	5.3E-01 (2.4E-02)	1.42 (0.24)	2.8E-01 (7.5E-01)	**2.43 (1.90)**	**3.0E-07 (2.6E-11)**	−0.08 (− 0.15)	4.7E-01 (3.5E-02)
Others	481 (1563)	41.4 (21.2)	53.0 (53.9)	12.1 (10.5)	−0.82 (−2.88)	8.3E-01 (2.1E-01)	−1.13 (− 0.02)	4.3E-01 (9.8E-01)	− 0.28 (1.06)	5.9E-01 (4.5E-04)	0.02 (− 0.10)	8.4E-01 (1.8E-01)
Qigong	545 (1589)	36.0 (22.3)	58.7 (59.8)	7.7 (6.5)	−3.63 (−4.43)	3.2E-01 (5.5E-02)	−1.88 (−2.71)	1.7E-01 (6.8E-04)	0.47 (0.44)	3.3E-01 (1.5E-01)	−0.13 (− 0.10)	2.5E-01 (2.0E-01)
Swimming	742 (1287)	67.0 (46.5)	52.2 (54.2)	11.0 (10.4)	−7.95 (−5.85)	1.2E-02 (2.2E-02)	−2.83 (1.14)	1.6E-02 (2.0E-01)	**2.48 (1.85)**	**5.0E-09 (2.6E-08)**	−0.24 (− 0.20)	1.3E-02 (1.7E-02)
Tai Chi	630 (1286)	52.5 (32.8)	57.0 (58.7)	8.9 (6.7)	−4.13 (−4.38)	2.3E-01 (8.7E-02)	−3.86 (−0.89)	2.3E-03 (3.1E-01)	0.29 (0.19)	5.3E-01 (5.7E-01)	−0.11 (− 0.08)	3.0E-01 (3.6E-01)
Weight training	383 (953)	76.2 (54.2)	43.2 (43.8)	10.9 (11.0)	0.54 (−9.44)	9.0E-01 (1.7E-03)	0.99 (1.65)	5.4E-01 (1.1E-01)	1.49 (1.56)	1.2E-02 (6.7E-05)	0.03 (−0.31)	8.4E-01 (1.2E-03)
Badminton	292 (561)	79.5 (52.0)	46.3 (47.7)	10.0 (10.8)	−5.39 (−0.15)	2.8E-01 (9.7E-01)	0.96 (0.06)	6.0E-01 (9.7E-01)	2.20 (2.08)	9.7E-04 (3.1E-05)	−0.22 (− 0.06)	1.5E-01 (6.4E-01)
Table tennis	262 (488)	78.2 (64.5)	54.3 (56.8)	10.6 (8.3)	−8.66 (−9.32)	1.0E-01 (2.3E-02)	0.71 (−1.95)	7.2E-01 (1.7E-01)	1.18 (1.82)	9.4E-02 (6.9E-04)	−0.24 (−0.28)	1.4E-01 (3.6E-02)
Basketball	191 (296)	96.9 (93.9)	41.0 (42.2)	9.8 (10.1)	−0.28 (6.59)	9.6E-01 (2.1E-01)	−1.30 (− 1.35)	5.7E-01 (4.6E-01)	0.40 (0.97)	6.3E-01 (1.6E-01)	0.01 (0.11)	9.6E-01 (5.1E-01)
Swing hands exercise	146 (329)	50.0 (28.3)	56.9 (58.2)	8.6 (7.6)	−1.62 (−5.63)	8.2E-01 (2.6E-01)	−2.48 (−1.48)	3.4E-01 (3.9E-01)	−1.50 (−0.25)	1.1E-01 (7.0E-01)	−0.01 (− 0.15)	9.6E-01 (3.6E-01)
Tennis	153 (267)	81.0 (78.3)	54.2 (55.9)	10.1 (9.3)	−13.74 (− 19.04)	4.6E-02 (5.9E-04)	− 1.07 (5.25)	6.7E-01 (6.1E-03)	3.24 (2.51)	4.1E-04 (5.0E-04)	−0.52 (− 0.58)	1.6E-02 (1.1E-03)
Yuan-ji dance	125 (289)	8.8 (4.2)	61.2 (61.3)	6.0 (5.5)	2.47 (−5.63)	7.4E-01 (2.9E-01)	−1.45 (−2.28)	6.0E-01 (2.2E-01)	−0.95 (0.60)	3.5E-01 (3.9E-01)	0.07 (−0.15)	7.7E-01 (3.8E-01)
Other ball exercise	76 (198)	76.3 (59.1)	47.1 (52.6)	12.4 (11.1)	−13.28 (−3.83)	1.7E-01 (5.5E-01)	2.24 (2.10)	5.3E-01 (3.4E-01)	2.72 (1.02)	3.6E-02 (2.2E-01)	−0.53 (−0.13)	7.7E-02 (5.2E-01)
Golf	111 (154)	85.6 (87.7)	54.0 (57.2)	9.2 (8.7)	−2.91 (−10.58)	7.2E-01 (1.5E-01)	3.47 (0.17)	2.4E-01 (9.5E-01)	0.49 (−0.16)	6.5E-01 (8.7E-01)	0.05 (−0.24)	8.5E-01 (3.0E-01)
Wai-tan kung	85 (162)	43.5 (24.1)	60.8 (61.1)	6.9 (5.5)	−3.61 (−5.52)	6.9E-01 (4.4E-01)	−7.05 (−2.92)	3.7E-02 (2.3E-01)	−1.19 (−1.26)	3.3E-01 (1.7E-01)	0.01 (−0.14)	9.6E-01 (5.3E-01)

Data are given for the discovery cohort (replication cohort). An association with *p* < 0.0004 = 0.05/(30 × 4) in both the discovery cohort and the replication cohort was highlighted in green

Table 2 shows the associations of 6 covariates with each dyslipidemia index, respectively. As mentioned above, a *p*-value less than 0.0004 is considered significant throughout this work. Female had a lower mean TG by 18.5 mg/dL (95% confidence interval [C.I.] = 16.2–20.8) and a higher mean HDL-C by 8.2 mg/dL (95% C.I. = 7.9–8.5) than males. Elder subjects had increased TG and LDL-C levels than younger subjects, but no significant association of age with HDL-C was observed (*p* = 0.13 and 0.04 in the discovery cohort and the replication cohort, respectively).

A strong association was found in BMI and all indices. A larger BMI was associated with increased TG, and LDL-C levels, and a decreased HDL-C level. Alcohol drinking was associated with elevated TG and HDL-C levels, and a decreased LDL-C level. The relationship between alcohol consumption and health outcomes remains controversial. On the contrary, cigarette smoking is consistently adverse to health by being associated with increased TG and LDL-C levels, and decreased HDL-C level. A higher education attainment was associated with a lower TG level.

### Associations of performing exercises with dyslipidemia indices

**Fig. 1:**
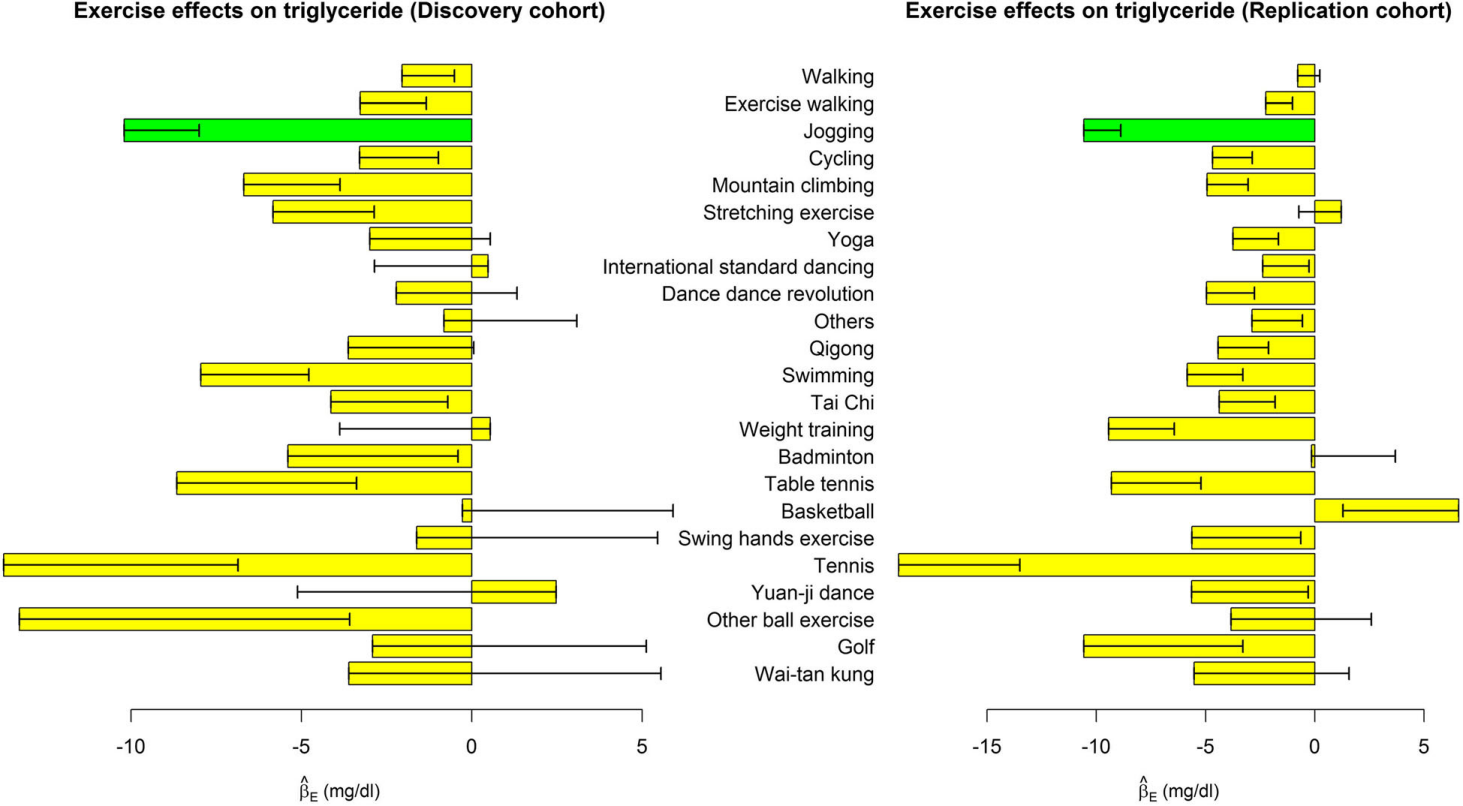
Exercise effects on TG. The regression model was built as $TG={\beta}_0+{\sum}_{j=1}^{23}{\beta}_{E_{j,k}}{E}_j+{\sum}_{v=1}^6{\beta}_{C_v}{Covariate}_v+\varepsilon$. The bars represent ${\hat{\beta}}_E$, and the black segments mark the standard error of ${\hat{\beta}}_E$. Covariates adjusted in the regression model included sex, age, BMI, drinking status, smoking status, and educational attainment. Bars with *p* < 0.0004 = 0.05/(30 × 4) in both the discovery cohort and the replication cohort were highlighted in green color

**Fig. 2:**
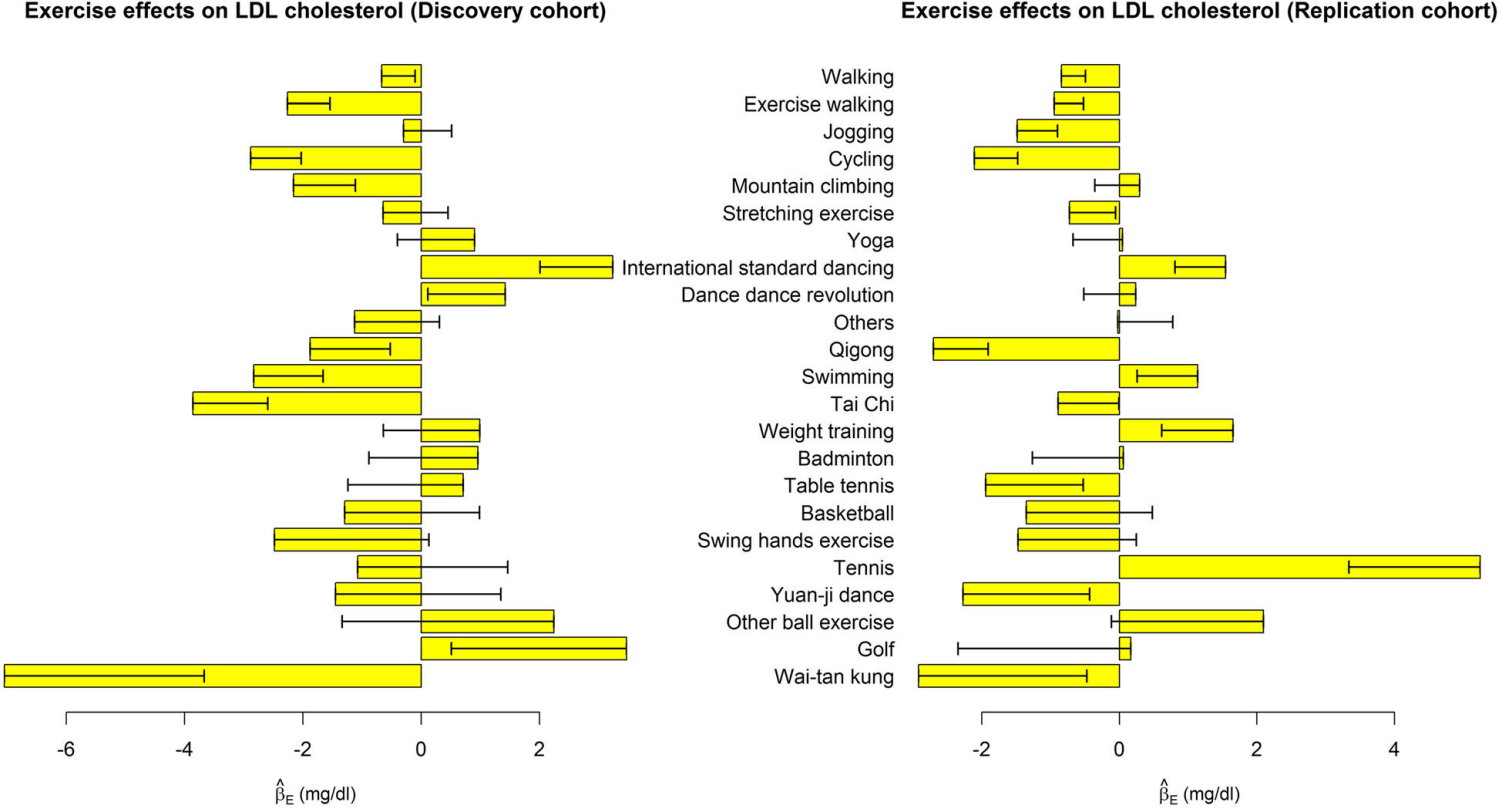
Exercise effects on LDL-C. The regression model was built as $LDL-C={\beta}_0+{\sum}_{j=1}^{23}{\beta}_{E_{j,k}}{E}_j+{\sum}_{v=1}^6{\beta}_{C_v}{Covariate}_v+\varepsilon$. The bars represent ${\hat{\beta}}_E$, and the black segments mark the standard error of ${\hat{\beta}}_E$. Covariates adjusted in the regression model included sex, age, BMI, drinking status, smoking status, and educational attainment. Bars with *p* < 0.0004 = 0.05/(30 × 4) in both the discovery cohort and the replication cohort were highlighted in green color

**Fig. 3:**
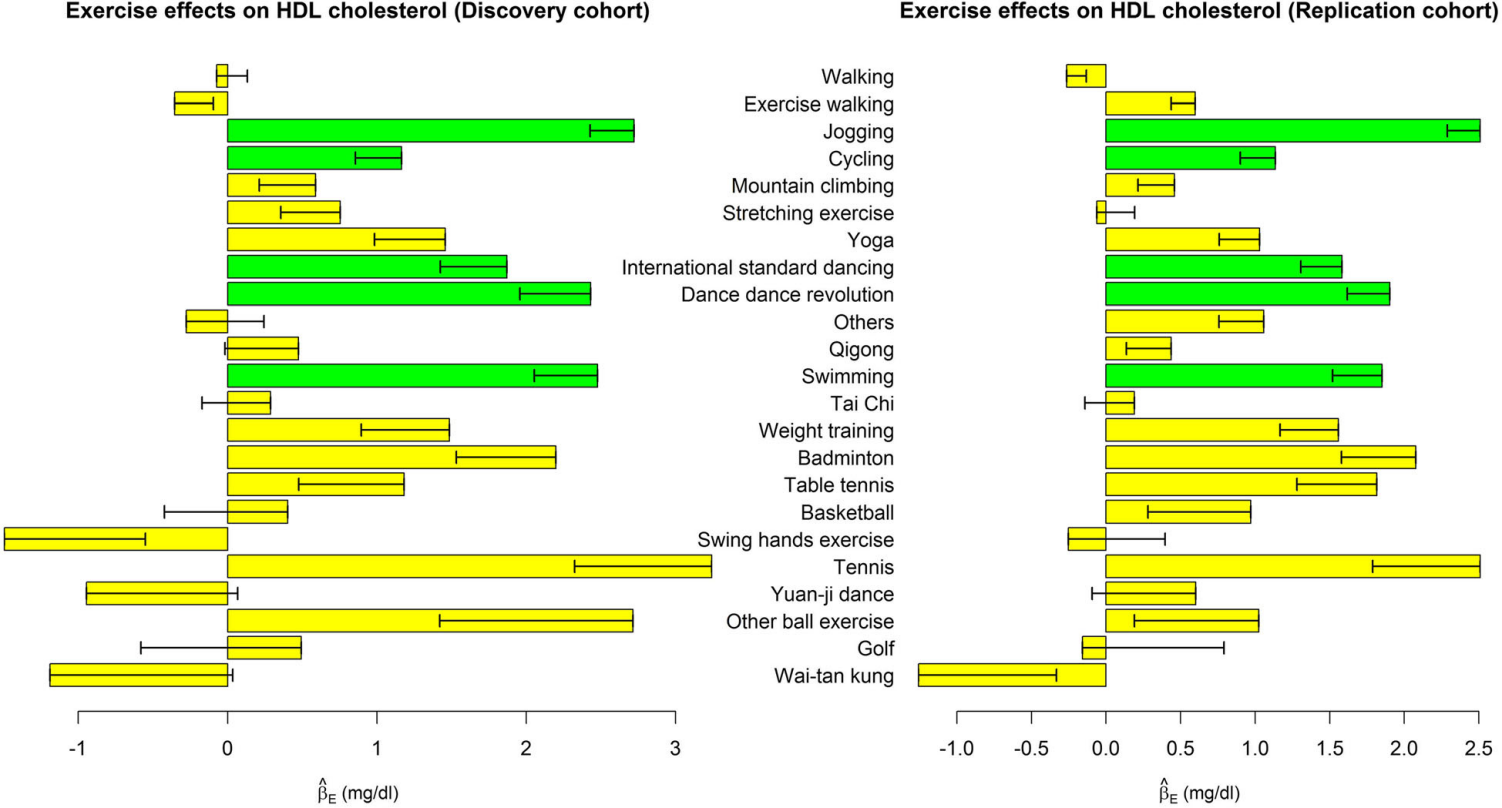
Exercise effects on HDL-C. The regression model was built as $HDL-C={\beta}_0+{\sum}_{j=1}^{23}{\beta}_{E_{j,k}}{E}_j+{\sum}_{v=1}^6{\beta}_{C_v}{Covariate}_v+\varepsilon$. The bars represent ${\hat{\beta}}_E$, and the black segments mark the standard error of ${\hat{\beta}}_E$. Covariates adjusted in the regression model included sex, age, BMI, drinking status, smoking status, and educational attainment. Bars with *p* < 0.0004 = 0.05/(30 × 4) in both the discovery cohort and the replication cohort were highlighted in green color

**Fig. 4:**
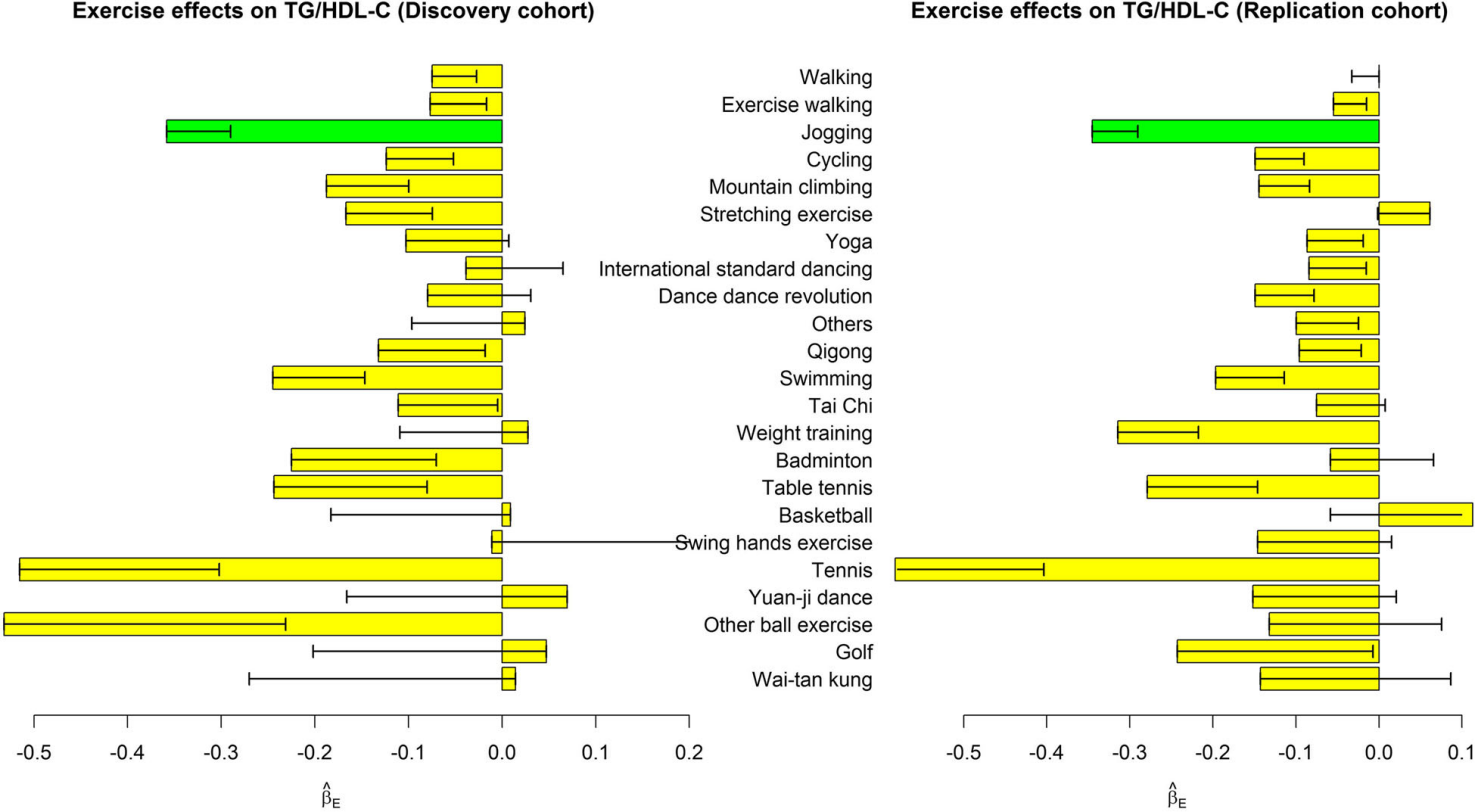
Exercise effects on TG/HDL-C. The regression model was built as $TG/ HDL-C={\beta}_0+{\sum}_{j=1}^{23}{\beta}_{E_{j,k}}{E}_j+{\sum}_{v=1}^6{\beta}_{C_v}{Covariate}_v+\varepsilon$. The bars represent ${\hat{\beta}}_E$, and the black segments mark the standard error of ${\hat{\beta}}_E$. Covariates adjusted in the regression model included sex, age, BMI, drinking status, smoking status, and educational attainment. Bars with *p* < 0.0004 = 0.05/(30 × 4) in both the discovery cohort and the replication cohort were highlighted in green color

Table 3 shows the associations of performing each exercise with 4 dyslipidemia indices, respectively. We also presented the effect sizes (${\hat{\beta}}_{E_{j,k}}\ \mathrm{in}\ \mathrm{model}\ (1),\mathrm{where}\ j=1,\cdots, 23,k=1,\cdots, 4$) in Figs. 1, 2, 3 and 4. Performing exercise was generally associated with a decreased level in TG (Fig. 1) and an increased level in HDL-C (Fig. 3). However, no significant associations were detected between any exercise and LDL-C (Table 3 and Fig. 2).

Subjects choosing jogging as their regular exercise had a lower TG level by 10.2 mg/dL (95% C.I. = 5.9–14.5), a higher HDL-C level by 2.7 mg/dL (95% C.I. = 2.1–3.3), and a decreased TG/HDL-C ratio by 0.36 (95% C.I. = 0.22–0.49). Moreover, swimming (95% C.I. = 1.6–3.3), dance dance revolution (DDR) (95% C.I. = 1.5–3.4), international standard dancing (95% C.I. = 1.0–2.7), and cycling (95% C.I. = 0.6–1.8) were associated with an increased level in HDL-C. These significant findings were well replicated in the TWB2 cohort, and the effect sizes (${\hat{\beta}}_{E_{j,k}}\ \mathrm{in}\ \mathrm{model}\ (1),\mathrm{where}\ j=1,\cdots, 23,k=1,\cdots, 4$) were similar across the TWB1 and TWB2 cohorts (Table 3).

## Discussion

This is a large-scale observational study evaluating the associations of regularly performing physical exercises with lipoprotein-lipid profiles. In line with the previous studies [4, 19], we here also found that HDL-C is the most sensitive index to exercise among the 3 dyslipidemia measures. Performing regular exercise has been found to improve HDL-C functionality, including cholesterol efflux capacity, anti-oxidative, and anti-inflammatory properties [17]. Cholesterol efflux capacity is a measure of the ability for a subject’s HDL-C to extract cholesterol from macrophages [29]. Moreover, exercise elevates lipid peroxide transport function of HDL-C [17, 30]. Finally, exercise training increased the ability of HDL-C to protect endothelial cells from injury [31].

Existing evidence on LDL-C response to aerobic exercises remains contradictory and inconsistent [9–12]. A number of studies have concluded that exercise has little effect on LDL-C unless combined with dietary change and weight loss [4, 32–34]. In this work, we have not found any significant association between LDL-C and physical exercise (Fig. 2).

Some RCTs have investigated exercise effects on dyslipidemia [20]. RCTs are usually small to medium in sample sizes, say, from 12 to 111 subjects [7, 9–12, 21]. Moreover, the kinds of exercise examined in RCTs are typically limited [7]. For example, LeMura et al. compared aerobic exercise with “resistance exercise” (or “strength training”) [9]. In that study, aerobic exercise included cycling, walking, and jogging; resistance exercise indicated weight training [9]. In a meta-analysis evaluating the efficacy of aerobic exercises on HDL-C [7], 22 out of 25 RCTs investigated the effects of walking, jogging, or cycling. Among aerobic exercises, these three kinds of exercise were commonly studied.

In this study, all the 5 exercises found to be associated with a healthier lipoprotein-lipid profile (i.e., jogging, swimming, DDR, international standard dancing, and cycling, as shown in Fig. 3) were examples of aerobic exercise. Another type of exercise, resistance exercise such as weight training, has not been found to improve people’s lipoprotein-lipid profile [9]. In line with this previous finding [9], we here did not observe any significant association between weight training and the 4 dyslipidemia indices.

While resistance exercise aims at increasing strength of muscles, aerobic exercise increases oxygen consumption without significantly changing strength [35, 36]. Aerobic exercise increases the activity of lipoprotein lipase (LPL) [37, 38], an enzyme produced by skeletal muscles and adipose tissues [39]. LPL can reduce circulating TG in the bloodstream [40]. Among aerobic exercises, jogging is especially a full-body exercise involving many body parts. To jog, one’s arms swing, legs and feet run, shoulders and abdomen are also involved in this continuous movement. As an aerobic endurance exercise, jogging can more effectively boost oxygen consumption and blood circulation [41]. A jogging training lasting for 12 weeks has been shown to increase serum LPL concentrations [42], which further facilitates the clearance of circulating TG and elevation of HDL-C level [43].

A limitation of this work is that exercise intensity (usually monitored by the percentage of one’s maximum heart rate) could not be monitored as what was done in RCTs. Although this is an observational study and all signals were explained as associations, many of our results were consistent with the results from previous RCTs [9–12]. Moreover, this is the first large-scale observational study linking various kinds of physical exercise to people’s lipoprotein-lipid profile.

## Conclusion

Our significant findings here were identified by the discovery cohort of 27,735 subjects and further well replicated by the replication cohort of 67,512 subjects. Through our analysis, we showed that regular jogging was not only associated with an increased level of HDL-C, but also the only one exercise associated with a decreased level of TG and TG/HDL-C ratio. Nonetheless, jogging may be difficult to engage in for subjects with limited exercise capacity. We here found that swimming, dancing (including DDR and international standard dancing), and cycling are also significantly associated with an increased level of HDL-C. People who are seeking exercise to improve their lipoprotein-lipid profiles can have other choices now.

## Data Availability

The datasets analyzed during the current study are available from the Taiwan Biobank, https://www.twbiobank.org.tw/new_web_en/index.php

## Abbreviations

DDR: Dance dance revolution
HDL-C: High-density lipoprotein cholesterol
LDL-C: Low-density lipoprotein cholesterol
RCT: Randomized controlled trial
TWB: Taiwan Biobank
TG: Triglyceride
